# Fecal calprotectin levels used as a noninvasive method for screening for chronic gastritis in pediatric patients. A descriptive study

**DOI:** 10.1590/1516-3180.2020.0765.R1.0904221

**Published:** 2021-08-13

**Authors:** Fatma Demirbaş, Gönül Çaltepe, Hasan Abbasguliyev, Ayhan Gazi Kalaycı

**Affiliations:** I MD. Physician, Department of Pediatric Gastroenterology, Hepatology and Nutrition, Ondokuz Mayis University Faculty of Medicine, Samsun, Turkey.; II MD. Professor, Department of Pediatric Gastroenterology, Hepatology and Nutrition, Ondokuz Mayis University Faculty of Medicine, Samsun, Turkey.; III MD. Postgraduate Student, Department of Pediatric Gastroenterology, Hepatology and Nutrition, Ondokuz Mayis University Faculty of Medicine, Samsun, Turkey.; IV MD. Professor, Department of Pediatric Gastroenterology, Hepatology and Nutrition, Ondokuz Mayis University Faculty of Medicine, Samsun, Turkey.

**Keywords:** Child, Gastritis, Leukocyte L1 antigen complex, Childhood, Chronic gastritis, Fecal calprotectin

## Abstract

**BACKGROUND:**

Gastritis consists of inflammation of the gastric mucosa and is one of the main causes of dyspeptic symptoms in children.

**OBJECTIVE:**

To investigate the presence of inflammation by evaluating fecal calprotectin (FC) in children diagnosed with chronic gastritis.

**DESIGN AND SETTING:**

Descriptive study in Pediatric Gastroenterology Department of Ondokuz Mayis University Hospital in Turkey.

**METHODS:**

Between January 2016 and July 2018, FC levels were compared retrospectively in children with chronic gastritis (histopathology-based diagnosis), patients with inflammatory bowel disease (IBD) and healthy children.

**RESULTS:**

A total of 67 chronic gastritis patients (61.2% girls) with a mean age of 13.09 ± 3.5 years were evaluated. The mean FC levels were 153.4 μg/g in the chronic gastritis group, 589.7 μg/g in the IBD group and 43.8 μg/g in the healthy group. These levels were higher in chronic gastritis patients than in healthy individuals (P = 0.001) and higher in IBD patients than in the other two groups (P < 0.001). The FC level in the patients with chronic active gastritis (156.3 μg/g) was higher than in those with chronic inactive gastritis (150.95 μg/g) (P = 0.011). Among the patients with chronic active gastritis, the FC level was significantly higher in *Helicobacter pylori*-positive individuals than in negative individuals (P = 0.031).

**CONCLUSION:**

We confirmed the association between increased FC and chronic gastritis. Elevated FC levels may be seen in patients with chronic active gastritis. In order to be able to use FC as a screening tool for chronic gastritis, further studies in a larger study group are needed.

## INTRODUCTION

Gastritis, which consists of inflammation of the gastric mucosa against various factors, is one of the most important reasons for dyspeptic symptoms in children.^1^ Children with gastritis frequently experience abdominal pain and other clinical symptoms. The diagnosis of chronic gastritis depends on histopathological findings.^2^ It is defined as an inflammatory infiltrate in the lamina propria, within the epithelium and foveolar lumen.^3^ Defects of mucosal protective barriers and disruption of the balance between the mucosal barriers and acid and pepsin levels play a role in the pathogenesis of gastritis.^4^ Intense neutrophilic infiltration in chronic gastritis can lead to erosion of the cytoplasm and destruction of epithelial cells. This can lead to mucosal ulcers that can cause tissue loss at levels ranging from the superficial to the submucosal layers.^2^

Fecal calprotectin (FC) is a protein that is secreted from macrophages and neutrophils. It is used quantitatively to show intestinal inflammation.^5^ It is 83% sensitive and 84% specific for distinguishing between organic and nonorganic diseases.^6^ FC levels are high both in inflammatory bowel disease (IBD) and in non-IBD diseases, including in microscopic colitis, infectious colitis, cystic fibrosis, celiac disease and nonsteroidal anti-inflammatory drug-induced enteropathy, which cause increased macrophage and neutrophil levels in the intestinal mucosa.^7^^–^^12^

## OBJECTIVE

Fecal calprotectin is widely used in diagnosing and following up IBD, but there are insufficient numbers of studies showing the relationship between FC and other upper gastrointestinal system diseases. The main objective of the present study was to compare the FC levels in children with chronic gastritis (based on histopathological findings) with those in healthy children and children with IBD; and to determine the factors affecting the FC level.

## METHODS

### Study design

Between January 2016 and July 2018, in the Department of Pediatric Gastroenterology, Hepatology and Nutrition of our institution, 573 patients underwent endoscopy. These patients had come to our clinic with a complaint of chronic abdominal pain. In addition to physical examination and laboratory tests, their fecal calprotectin level was determined and abdominal ultrasound and abdominal X-ray were performed. Interventional procedures such as upper endoscopy and colonoscopy were performed in order to investigate the underlying etiology. Sixty-seven patients who were diagnosed with chronic gastritis (through histopathology-based diagnosis) and whose FC levels were assessed were included in this study.

The following individuals were not included in this study: patients for whom FC was not tested; patients who were not diagnosed histopathologically as having chronic gastritis; patients with upper endoscopy or pathology-verified mucosal lesions such as esophagitis, gastric ulcer, gastric polyp or duodenal ulcer; patients with chronic diseases; patients reacting positive for acute-phase disease; patients who were positive for infection in stool samples; patients with a history of drug use; and patients with a history of chronic disease.

The patients’ demographic characteristics, physical characteristics (height Z score and weight Z score) and laboratory findings (white blood cells, thrombocytes, C-reactive protein (CRP), erythrocyte sedimentation rate (ESR), albumin and hemoglobin) were recorded.

### Endoscopy procedure

All the endoscopy procedures were performed by an experienced gastroenterologist. Gastric antrum, gastric corpus and incisura angularis biopsies were obtained from the patients during endoscopy. Patients with chronic gastritis were classified in accordance with the Sydney classification.

Hematoxylin-eosin preparations were initially used to examine the biopsy samples for the presence of *Helicobacter pylori.*^13^ When the hematoxylin-eosin preparations revealed organisms in a biopsy specimen, situations of chronic active inflammation was then detected by using the modified Giemsa and toluidine blue stains. Chronic active gastritis was taken to be the presence of a mixed inflammatory infiltrate in the lamina propria, in the presence or absence of *Helicobacter pylori* organisms. Chronic inactive gastritis was taken to be the presence of dense populations of lymphocytes and plasma cells within the lamina propria, in the presence or absence of *Helicobacter pylori* organisms.^14^

There were two control group: one that included patients with IBD at the time of diagnosis by means of upper and lower endoscopy and the other that comprised healthy controls of the same ages as the patients, without any chronic disease or signs of infection.

### Fecal calprotectin measurement

Three fecal specimens were collected from each child at least one week before endoscopy. These stool specimens were stored at 2-8 °C until tested. The FC evaluation kit was analyzed in accordance with the manufacturer's instructions (RIDA TUBE Calprotectin; R-Biopharm AG, Darmstadt, Germany). FC levels ≤ 50 μg/g were accepted as normal, and levels > 50 μg/g as abnormal.

### Ethics

This study was approved by the ethics committee of our hospital (Clinical Research Ethics Committee decision number 2018/139; date: March 30, 2018. Written informed consent for all procedures was obtained from the parents or legal guardians of each child in the study.

### Statistical analysis

The statistical analyses were performed using the Statistical Package for the Social Sciences (SPSS) software, version 22.0 (IBM Corporation, Armonk, New York, United States). The data were presented as the mean ± standard deviation, number (n) and percentage (%). The Shapiro-Wilk test was used to analyze the normal distribution assumption of the quantitative outcomes. Comparisons among the independent binary groups with normal distribution were made using Student's t test, and the analysis of variance (ANOVA) test was applied to hypervariable groups. The Mann-Whitney U test was used to compare pairs of groups of data that did not show normal distribution, and for over-variant groups. To compare the percentages of the qualitative data, the paired chi-square test and z-test were applied. P-values < 0.05 were considered statistically significant.

## RESULTS

A total of 67 children with chronic gastritis were included in the study, comprising 41 girls (61.2%) and 26 boys (39%). Their mean age was 13.09 ± 3.5 years (range, 5-17.9).

All the patients were older than four years and they were all negative for stool infectious markers. The patients included had no evident history of any other disease, usage of any drugs, or positivity for inflammatory markers such as CRP and ESR.

Regarding the histopathological findings from the patients according to the Sydney classification, 38 patients (56.7%) had chronic active gastritis, 29 (43.3%) had chronic inactive gastritis, 28 (41.7%) had *Helicobacter pylori*-positive gastritis (n = 16 with chronic active gastritis and n = 12 with chronic inactive gastritis) and 39 (58.2%) had *Helicobacter pylori*-negative gastritis (n = 22 with chronic active gastritis and n = 17 with chronic inactive gastritis).

The mean FC level in the patients with chronic gastritis was 153.4 μg/g (range, 19.5-550); chronic active gastritis, 156.3 μg/g (range, 109-550); and chronic inactive gastritis, 150.95 μg/g (range, 19.5- 250). The FC levels were higher in patients with chronic active gastritis than in those with chronic inactive gastritis (P = 0.011).

The FC levels were significantly higher in *Helicobacter pylori*-positive patients with chronic active gastritis (157.1 μg/g) than in *Helicobacter pylori*-positive patients with inactive gastritis (152.3 μg/g) (P = 0.024). There was no significant difference in FC levels between chronic gastritis patients who were *Helicobacter pylori*-positive (154.7 μg/g) and *Helicobacter pylori*-negative (151.1 μg/g) (P = 0.486). However, among chronic active gastritis patients, the FC level was significantly higher in those who were *Helicobacter pylori*-positive than in those who were *Helicobacter pylori*-negative (P = 0.031) (**Table 1**).

**Table 1:** Comparison of laboratory findings among patients with chronic active gastritis and chronic inactive gastritis

Total (n = 67)	Chronic active gastritis (n = 38)			Chronic inactive gastritis (n = 29)		
	HP (+) (n = 16)	HP (-) (n = 22)	P-value	HP (+) (n = 12)	HP (-) (n = 17)	P-value
Fecal calprotectin (μg/g)	157.1 (120-550)	153.4 (109-521)	0.031	152.3 (54-175)	149.6 (19.5-196)	0.423
C-reactive protein (mg/l)	9.4 ± 2.9	9.1 ± 3.1	0.416	7.8 ± 3.3	7.0 ± 2.8	0.343
Erythrocyte sedimentation rate (mm/h)	13.6 ± 3.2	12.9 ± 4.3	0.234	15.6 ± 3.8	15.2 ± 3.1	0.212
White blood cells (x 10^3^/ul)	6.6 ± 2.9	6.1 ± 2.3	0.214	5.8 ± 2.8	5.6 ± 2.4	0.112
Hemoglobin (g/dl)	12.8 ± 0.6	12.7 ± 0.3	0.323	13.4 ± 1.6	13.1 ± 1.4	0.314
Albumin (g/dl)	4.6 ± 0.7	4.1 ± 0.9	0.447	4.8 ± 1.4	4.1 ± 1.8	0.178
Thrombocytes (x 10^3^/ul)	386 ± 54	372 ± 46	0.349	329 ± 157	321 ± 168	0.456

HP = *Helicobacter pylori*.

Comparison of the gender, age, height Z score, body weight Z score, BMI Z score and laboratory findings from chronic gastritis patients showed that there was no statistically significant difference between FC levels > 50 μg/g (n = 42; 67%) and normal levels (**Table 2**).

**Table 2:** Comparison of demographic findings among patients with chronic gastritis

Total (n = 67)	FC > 50 μg/g (n = 42)	FC ≤ 50 μg/g (n = 25)	P-value
Age (year)	13.1 ± 4.08	13.1 ± 3.6	0.614
Gender (female/male)	27/15	14/11	0.606
Height Z score	−0.53 ± 0.9	−0.48 ± 0.8	0.696
Weight Z score	−0.63 ± 0.9	−0.53 ± 1.2	0.166
Hemoglobin (g/dl)	12.8 ± 0.5	13.0 ± 1.5	0.301
White blood cells (x 10^3^/ul)	6.4 ± 2.8	5.7 ± 2.9	0.120
Thrombocytes (x 10^3^/ul)	379 ± 46	325 ± 167	0.509
C-reactive protein (mg/l)	9.2 ± 3.2	7.4 ± 3.1	0.135
Erythrocyte sedimentation rate (mm/h)	13.1 ± 4.2	15.4 ± 3.5	0.872
Albumin (g/dl)	4.3 ± 0.5	4.5 ± 1.0	0.179

FC = fecal calprotectin.

Colonoscopy was performed later in all of the 42 children, in order to minimize false positivity of the test (due to IBD, drug intake or infectious diarrhea), and the results were found to be normal, both macroscopically and microscopically.

### Comparison with the control groups

Among the IBD patients (n = 20), 12 (60%) were female and the mean age was 11.5 ± 3.5 years (range, 5-17.6). Thirteen patients (65%) had ulcerative colitis and 7 (35%) had Crohn's disease. The FC level of the IBD patients was evaluated at the time of diagnosis and the result was found to be 589.7 μg/g (range, 19.5-800).

Among the healthy children (n = 20), 11 were males (55%) and the mean age was 10.5 ± 3.3 years (range 5-15.1). The mean FC level in healthy children was 43.8 ± 25.4 μg/g (range, 19.5-144). There was no difference between the gastritis, IBD and healthy groups in terms of age and gender.

**Figure 1:**
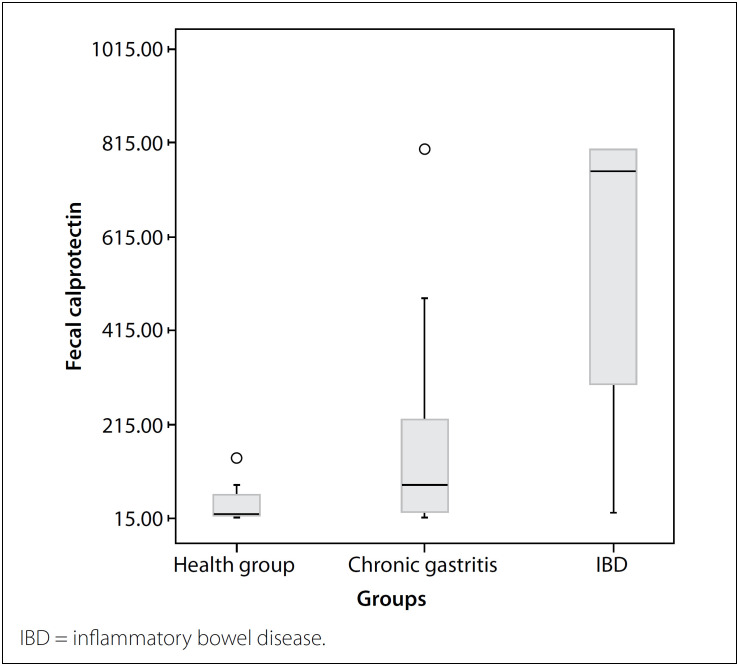
Evaluation of fecal calprotectin between groups.

The FC level in the children with chronic gastritis was significantly higher than in the healthy group. (P = 0.001). Compared with the IBD patient group, the FC level was found to be significantly lower in the children with the chronic gastritis and healthy group (P < 0.001) (**Figure 1**).

## DISCUSSION

Chronic gastritis is a chronic inflammatory process in the gastric mucosa and it is one of the most prevalent findings from endoscopy and histopathological examination.^15^^–^^16^ In the present study, 67% of the patients with chronic gastritis had high FC levels. There are only limited numbers of studies evaluating the relationship between chronic gastritis and FC.^17^^–^^19^ In the study by Manz et al.,^17^ conducted on a total of 147 patients, the FC level was higher in patients with erosive gastritis than in normal patients. However, Montalto et al.^18^ reported that there was no difference in FC levels between patients with gastritis (n = 61) (based on histopathological findings) and the healthy group.

Fecal calprotectin is a quantitative biomarker that detects inflammation of the gastrointestinal tract.^20^^–^^22^ In the present study, the FC level in chronic gastritis patients was significantly higher than that of the healthy group. Colonoscopy was performed later on among the chronic gastritis patients with high FC levels, and the macroscopic and microscopic evaluation of these patients were normal. None of the patients were positive for stool infection markers, histories of chronic systemic disease, acute-phase reactants or drug usage. This supports the notion that the FC levels in this study represented an expression of chronic gastritis as the result of mucosal inflammation. The FC level was higher in the IBD patients than in the chronic gastritis patients. The higher FC levels in the IBD patients may be explained by the occurrence of more excessive inflammation, of greater severity, in the gastrointestinal tract than in the chronic gastritis patients, whose inflammation was limited to the gastric mucosa alone.

In this study, the FC level in the children with chronic active gastritis was higher than the level in the children with chronic inactive gastritis. The presence of neutrophils due to chronic inflammatory cells indicates the presence of chronic active gastritis.^13^ The elevated FC levels in chronic active gastritis may be due to mucosal injury that is mediated by increased levels of mucosal macrophages and neutrophils. However, Montalto et al.^18^ did not find any significant difference in FC levels between patients with chronic active gastritis and those with chronic inactive gastritis.

*Helicobacter pylori* infection is commonly acquired in childhood and affects one third of all children worldwide.^23^ Its prevalence is low in developed countries but high in developing countries.^24^ The standard method for diagnosing *Helicobacter pylori* infection is endoscopic biopsy of the gastric antrum.^25^ In the present study, although there was no significant difference in FC levels between *Helicobacter pylori*-positive and *Helicobacter pylori*-negative patients, the FC levels of *Helicobacter pylori*-positive chronic active gastritis patients were significantly higher than those of *Helicobacter pylori*-negative chronic active gastritis patients. Sýkora et al.^19^ found that the FC levels of patients with *Helicobacter pylori*-positive gastritis were normal, in comparison with healthy children. Summerton et al.^26^ showed that in patients with upper gastrointestinal inflammation such as gastritis and duodenitis, their FC levels were normal. Additionally, in the present study, the FC levels were significantly higher in *Helicobacter pylori*-positive patients with chronic active gastritis than in *Helicobacter pylori*-positive patients with chronic inactive gastritis.

*Helicobacter pylori* infiltrates neutrophils, lymphocytes, monocytes, mast cells and eosinophils in the gastric mucosa.^13^ The reason why significantly higher FC levels were observed only in *Helicobacter pylori*-positive patients with chronic active gastritis may have been because of greater severity of tissue damage caused by *Helicobacter pylori* in chronic active gastritis, through inducing neutrophil activation. In agreement with the data presented in the literature, our results in this study showed that the gastric inflammation was correlated with the *Helicobacter pylori* infection.^27^

There are, however, some limitations to this study. While the relationship between histopathological findings and FC was evaluated, the relationship between the patients’ clinical symptoms and FC was not evaluated. Another limitation of this study was the low number of patients.

## CONCLUSION

We found higher FC levels in patients with chronic active gastritis than in patients with chronic inactive gastritis. Additionally, the FC levels were higher in patients with chronic gastritis than in the healthy group and lower than in the IBD group. In the chronic gastritis patients, inflammation in the gastric mucosa caused a significant increase in FC level, compared with the healthy control group. However, the increment in FC levels in the chronic gastritis patients was lower than that of the FC levels in the IBD group, due to the lack of widespread involvement of the gastrointestinal tract inflammation in chronic gastritis. In the children with chronic abdominal pain and high FC levels, treatment of gastritis can be done firstly and then, if the FC level remains the same or rises, endoscopy with colonoscopy can be performed. Further studies in a larger study group are needed in order to be able to use FC as a screening tool for chronic gastritis.
